# The First Men to Practise Dentistry in Boston

**Published:** 1903-07

**Authors:** Adelbert Fernald

**Affiliations:** Boston, Mass.


### THE FIRST MEN TO PRACTISE DENTISTRY IN BOSTON.[1]

BY ADELBERT FERNALD, D.M.D., BOSTON, MASS.

Mr. President and Gentlemen,—The subject which I have selected for this evening is one which, in order to make it interesting and instructive to you, requires more time and study than I have been able to give to it.

Many of you here to-night may not care to know what the den-

[1] Read before the Harvard Odontological Society, December 18, 1902.

tists did one hundred years ago, but would much prefer to hear what is being done to-day.

In order to understand and appreciate the great advancement in art and science in any line, it is necessary that we know something about its history. We can then make comparisons and can see at a glance where the improvement lies; and what, may I ask, has made any more advancement, what standard has been raised any higher than the dental profession? When we stop and think of Wells, of Morton, and what they did to relieve suffering humanity, to say nothing about numerous other men, we may feel proud that we are dentists and have had such men in our calling.

The dental profession has done much—it will do more. The more we know about its history and the hardships of the first men to practise, the more inspired we shall all be, and strive to lift our standard higher.

It is not my intention to-night to give a complete history of the beginning of dentistry in this country, but simply to read and show you a few things which I am in hopes will throw "a side-light on it." Dentistry had its beginning right here in Boston.

The earliest record we have of dentistry in this country is in 1636, when three barber-surgeons came to Boston from London. The name of one only, William Dinley, is known.[1] In 1639 a Roxbury man suffering from toothache sent for him to come and extract the tooth. He started on his mission of mercy accompanied by a maid who brought the message. They were overtaken by a violent snowstorm, lost their way, and were found some days after, frozen and dead. His misfortune has preserved his name to posterity. Madam Dinley shortly after gave birth to a son, who was named Fathergone Dinley.[2]

A New York paper, published in 1766, contains this advertisement: "James Daniel, wigmaker and hair-dresser, also operator upon the teeth." [3]

The next man that I have any record of is Mr. John Baker, surgeon-dentist from England, of whom little is known. He instructed Paul Revere in dentistry, and in May, 1768, he moved to New York, turning his practice over to Revere.

---

[1] Memorial History of Boston, Justin Winsor, 1881, vol. i. p. 502.

[2] Annals of New York City and State, John F. Watson, 1846, p. 281.

[3] Trueman's Dental Science in the United States.

Born January 1, 1735, Paul Revere was, at this date, 1768, a young man beginning to make his reputation as engraver and still following his trade as a goldsmith. He then took up the method of replacing lost teeth by artificial ones. An expert worker in gold and silver, he could easily turn his skill to dental art. There is now in the Art Museum a silver tea-set made by Paul Revere which, I am afraid, no dentist to-day would or could duplicate. The following is one of his dental advertisements:

> "He flatters himself that from the experience he has had these two years (in which time he has fixed some hundreds of teeth) that he can fix them as well as any Surgeon Dentist who ever came from London. He fixes them in such a manner that they are not only an ornament but of real use in Speaking and Eating: He cleanses the Teeth and will wait on any Gentleman or Lady at their Lodgings, he may be spoke with at his shop opposite Dr. Clark's."

His artificial teeth are said to have been carved from ivory and fastened with gold wire. There is no evidence to show that he filled cavities. After the evacuation of Boston by the British, in March, 1776, the friends of General Warren were informed where the latter had been buried in the grave with a person "with a frock on." Identification was rendered doubly certain by Colonel Revere, who set the artificial tooth and who recollected the wire he used in fastening it.[1] Paul Revere died in Boston, May 10, 1818.

"In 1650 Nathaniel Greenwood came to Boston from Norwich, England, and engaged in business as a ship-carpenter. He died in 1685, leaving two sons, Samuel and Isaac. The latter became the first professor of mathematics at Harvard College, and was the father of Isaac, Jr., whose name is first brought to notice in a newspaper account of the Boston Massacre, March 5, 1770. He is there described as an ivory turner, 'a business naturally embracing that of a dentist.' He is said by Dr. C. A. Harris to have been the 'first practical dentist in Boston.'"[2]

I have here the *Massachusetts Sentinel,* published in Boston, 1789, which contains Isaac Greenwood's advertisement, which I will pass around.

> "He practised in Boston about twenty years. He had five sons; three of them became dentists. Two, John and his younger brother Wil-

---

[1] Psi Omega Dental Journal, Boston, 1897, vol. i. p. 14.

[2] Trueman's Dental Science in the United States.

liam Pitt, will ever be remembered for their good work, John becoming dentist to President Washington. William Pitt Greenwood was an ivory turner, and after studying for a short time in New York with his brother John, he returned to Salem and practised as a dentist in 1790; later he came to Boston and practised in this city until disabled by old age. He died in Boston, 10th of May, 1851, at the age of 85. Dr. William Pitt Greenwood was very skilful in carving bone teeth; he had a good reputation, and enjoyed a large practice. In 1840 he received an honorary degree of D.D.S., from the Baltimore Dental College."

Before I say anything more about dental science, I wish to call your attention to Boston at this time. I have here a reproduction, which I will pass around, of Tremont, Winter, and School Streets, where some of the early dentists had their offices. In 1790 the population of Boston was eighteen thousand. There were twenty-three physicians and three or four dentists. The law at this time was very severe, a woman being hung for stealing a bonnet valued at five shillings. On Colonnade Row, now Tremont Street, Dr. John T. Codman's father saw a man in the pillory, with hands, head, and feet yoked, and the boys pelting him with rotten eggs. If a man was poor and could not pay his debts, he was not allowed to live within the town limits, but could live outside of the limits, and if caught inside, no matter in what peaceful pursuit engaged, could be lodged in jail and kept there until his debts were paid. However, any man could practise dentistry if he could get any one for a patient. One of the early dentists [1] says "that when he began practising the word *dentist* was hardly heard." Some men made teeth for themselves. Mr. L. Pool (whose brother invented Pool's index system) told me that his father carved for himself, from a whale's tooth, set after set as he needed them. When Dr. John T. Codman was a young man [2] the boys here in Boston went to the constable, a Mr. Clapp, to have their teeth extracted. When a man wished to become a dentist a hundred years ago he did not have a dental school to go to. Books on the subject were few and very hard to obtain. Those who were practising were careful to keep all they had. One could not obtain any information from them without paying a large sum. Sometimes a man would be taken into an

---

[1] Extracts from Dr. John T. Codman's address before the American Academy of Dental Science, November 13, 1889.

[2] William P. Greenwood, American Journal of Dental Science, vol. iii. p. 77.

office for one thousand dollars[1] and taught mechanical dentistry, as that, to a limited extent, was all that was done. Most all instruments were made, perhaps, in a blacksmith's shop, roughly, to be sure, but still much was accomplished. By learning to make such instruments or appliances as were needed, they became used to doing fine mechanical work.

The *Boston Gazette,* December 22, 1777, contains this advertisement: "A few surgical and dental instruments for sale. The lowest price for same two hundred and eighty continental dollars."

Many of the first men were skilful in the use of tools, such as carpenters, machinists, and jewellers, the latter being skilful in working gold and silver and in doing fine soldering. A great many tools they used in their work were just what was needed in dental work, such as pliers, drills, saws, files, and polishing implements. At this time a man was greatly handicapped if he wished to take up dentistry unless he had some experience in the jewelry line.

I find in the *Massachusetts Spy,* January, 1773, the advertisement of Daniel Scott, a Boston dentist, and in the *Massachusetts Gazette,* January 27, 1774, the following:

> "Dr. Spencer, dentist, Temple St. Teeth extracted after an easier method than hitherto practised."

The first Boston directory published, 1789, gives the names of three dentists,—John Templeton, Isaac Greenwood, and Josiah Flagg. I find in the *Sentinel,* September 20, 1786, and May 27, 1789, John Templeton, Dentist, advertises to pay cash for live teeth to any person who would part with them. About this time M. Le Mayeur, a dentist, advertises in a Philadelphia paper, 1784, to give two guineas, or about ten dollars, each for sound teeth to those who would sell them. The *Columbian Sentinel,* 1790–91, contains these advertisements which, I presume, are father and son:

> "Josiah Flagg, Sr., a jeweller, try me, prove me, hold fast that which is good. Josiah Flagg, Jr., Surgeon Dentist, has the honor to acquaint the ladies and gentlemen of the metropolis and vicinity that he has removed from Milk St. and taken the stone house of Mr. J. Allen's, the second house on the right hand going up Beacon St. from the Stone Chappel where he has every convenience for his practice. He alleviates the most acute pain without the use of instruments. Stops hæmorrhage arising from any cause whatever. Cleans the teeth and gums, restoring

[1] History of Dental and Oral Science in America.

them free from future injury. Makes artificial teeth with and without roots. The former equally as serviceable as the natural ones. Practises the various branches of the dental art upon a new, much improved, highly recommended and really safe method. Cash given for live teeth to no person but such as are in perfect health. The poor ye have always with you, they are cheerfully provided assistance gratis."

Josiah Flagg was born September 30, 1763. Of his early life but little is known. No doubt he inherited from his father his great mechanical skill and ingenuity. We know that he was patriotic, enlisting in the army when only eighteen. He became a major, and while the French and American armies were in winter quarters in Rhode Island, 1781–82, he had an opportunity to study dentistry under Joseph Lemarie, a French officer and surgeon. Serving until the close of war, he shortly began to practise as a dentist in Boston. In 1812, when this country was again in war, he enlisted in the navy, but was shortly captured and taken to England, where he was paroled. His reputation as a dentist introduced him to some of the noted surgeons of London. (In fact, he was received with much honor),[1] and is said to have assisted Sir Ashley Cooper at Guy's Hospital. By attending the lectures and associating with such men, he acquired great skill in operative as well as mechanical dentistry. And if he did the surgical operations which he advertised to do, such as sewing up harelips, he did what many of us to-day do not. He was one of the first to fill teeth with soft gold. So soon as the war was over he returned to this country, ready to give his friends and the public the benefit of his knowledge. His health failing soon after, he went to Charleston, S. C., where he died September 30, 1816.[2] We have records of two sons who were noted dentists,—Dr. J. F. Flagg (whom I will refer to later on) and Dr. J. F. B. Flagg, who practised in Philadelphia.

We think of dentistry at this time as being very limited, yet considerable was accomplished. Teeth were extracted, decay filed away, and cavities sometimes filled with lead-foil and other gummy substances. Soft gold was also used about this time, but tin not until 1822. Teeth were filled until one-fourth or even one-third was cut away. Not until 1822 do we find any objections to this practice. Josiah F. Flagg recommended using crooked knives and

[1] Dental News Letter, vol. vii. p. 212.
[2] Ibid.

sharp instruments. As late as 1836 Dr. Spooner used files to cut away decay, but did not separate sound teeth as did the earlier dentists. He says, " Do not file a tooth that can be better and more effectually treated by plugging." The art of filling teeth in 1789 was not practised to any great extent. Loose teeth were treated and cleaned. Very little regulating was done, and that mostly by extracting, although teeth were ligated to place in some cases. Benjamin James, in 1814, says, " Irregular teeth in young persons are to be pressed to place by the fingers." Most of the work was to replace teeth, when lost, by carving and with bone plates. Sometimes the plates were carved from a sea-horse's or a whale's tooth. They had no cement, no rubber dam, no engines, no vulcanite, no amalgam, no cohesive gold,—not even a match, friction matches not being invented until 1827,—no coal gas, no nitrous oxide, ether, or chloroform. Soldering was done by a spirit- or oil-lamp. When a plate was made, a section of bone or tooth was cut so as to have the enamel come in front of the carved tooth. Often the plate was carved first, and six or eight human teeth were attached in front by means of silver and gold screws or wooden pegs. Often the teeth were made of oxen's, horses', and cows' teeth, ivory, mother-of-pearl, and wood. Of all animal substances for artificial teeth, the human tooth was the best. I will give a brief description of how these plates were made. An impression was taken in wax, " wax being held at first by the fingers and a model was made with plaster of Paris," plaster being used for this purpose for ages. A piece of bone a little larger than the model was selected, the model painted or colored with a gummy preparation so as to leave a mark on the bone, then the spots were carved away, this being repeated until the whole surface of the bone became colored; then it was supposed to fit. Sometimes bone plates had the gums stained to give a more natural color. If it was a partial plate it was often ligated to place by silk cord, cat-gut, or platinum wire. If a whole set, spiral springs were attached. You will get a much better idea from these specimens, which I will pass around. On one of them pieces of the old cat-gut or fiddle-string still remains which held the block in place. For crowning a tooth at this time, " The fee was from $3 to $10." [1] These bone teeth or plates were not very durable, unsightly, discoloring very shortly after being put into

---

[1] Benjamin James.

the mouth, and were apt to decay. In 1795 Dr. John Greenwood, in a letter to his distinguished patient, George Washington, tells him to fill the cavities in his bone plates by drying the same and melting sealing wax into the cavities with a hot nail.

The Boston directory of 1804 contains the names of three dentists,—William Pitt Greenwood, Cold Lane; Josiah Flagg, Black Street; Thomas Parsons, 1 Winter Street.

In the *Columbian Sentinel,* February 9, 1805, we find the following:

> "T. Parsons, Dentist, Respectfully informs the inhabitants of Boston and vicinity that he continues to practise in the line of his profession."

John Randall, M.D., practitioner of medicine and dental surgery, was born in Stow, Middlesex County, in 1773; graduated at Harvard in class of 1802; studied medicine with the celebrated Dr. John Jeffries, of Boston, and commenced practice about the year 1805. During his collegiate course he found his own teeth beginning to decay, and sought relief of the best dentists of that day, who told him that his business was to put in new teeth, and declined any operation for the preservation of the natural ones. Dr. Randall commenced his first dental operations upon himself. While a pupil in medicine he performed many operations for his classmates. He never advertised; his modest announcement to the world was "Dr. John Randall." Dr. Randall was very successful and popular. In extracting teeth he used the key, also the forceps, with great skill. He was scrupulously conscientious; he was decidedly conservative in his theory and practice of dental surgery. He knew the value of saving healthy roots for crowns; his success in engrafting teeth was very great. For nearly forty years he was a useful and honorable member of society, and bestowed much upon the poor and accumulated ample provision for his family. He died in 1843, aged seventy years.

Teeth were crowned by cutting off and burning out nerve, or, as Benjamin James says, "When the nerve is alive, to remove it take an instrument the size of a brown thread needle and carry into the natural canal of the root as far as it will go. The remainder of this operation will be free from pain." Arsenic was not used for this purpose until 1836 by Dr. S. Spooner. Sometimes the crown was set with a gold or silver screw, often by a hickory post, in some cases cotton being wrapped around the pivot to make it stay in place.

Dr. Josiah Foster Flagg was born in Boston, January 10, 1788. When a small boy he showed great skill and enjoyment in work. When he was sixteen years of age he turned his attention to cabinet-making and shortly after developed a strong desire for study. Then he was sent to an academy at Pittsfield, Conn. He took up the study of medicine and surgery with Professor J. C. Warren, who very quickly discovered his genius and ability. After his graduation Dr. Flagg located as a practising physician and surgeon in Dover, N. H., and Uxbridge, Mass., remaining in each about two years. In 1819, the year after his marriage to Miss Mary Waite, Dr. Flagg removed to Boston, and gave his entire attention to dentistry. In 1830 he excelled all in the making of artificial teeth. When we consider the fact that he possessed great skill in the use of the knife as a dissector, and often constructed surgical instruments and anatomical preparations and methods for alleviating pain, and had frequently assisted his father in mechanical operations of dentistry, we can see how he might succeed. All this being known, it was quite sufficient at that time for the people of the community to put their trust and confidence in Dr. Flagg. And here, in Boston, with a large circle of patients and loyal friends he practised the profession for thirty-four years. Dr. Flagg contributed freely to the list of inventions and suggestions which have helped to relieve suffering humanity, among which were his wax or triangle splints for fractured limbs and a great variety of bone-forceps, which have now very much superseded the use of the saw in certain amputations, and in 1828 he invented forceps which were calculated to apply to every variety of teeth. Dr. Flagg was an artist of no mean ability, and founded the first school of design in this country. He died in Boston, July 20, 1854.

I have here some turnkeys which I will pass around. The key of Garengeot was invented in the early part of the eighteenth century for the extraction of teeth. It is an improvement on the ancient pelican. Since the time of Garengeot the key has undergone a number of changes. The first material improvement was made by Mr. Spence. It consisted of adding a projecting part at the end of the bolster, and through which the screw is passed. This addition was for the purpose of fixing a claw in an advanced position beyond the bolster, which was found extremely useful in the extraction of the wisdom-teeth. The round fulcrum and raised shaft are improvements made by Mr. Savigny, and more recently Duval added the movable bolster, but none of the above improvements have contributed very materially to the value of the instrument except the round fulcrum. The key, notwithstanding all the improvements, has fallen into disuse and is superseded by the forceps. The forceps for extracting teeth was probably among the first, perhaps almost the only instrument employed for the extraction of teeth until the invention of the key by Garengeot.

Oliver Wendell Holmes says, "There never was a claw on bird or beast that was the cause of such anguish of apprehension, such howls of agony, as that diabolical instrument, looking like a vulture's talon, but known as the key. It was a key indeed. It may have opened the door of heaven to the sufferer in due time, but while the bolt was turning the victim thought he was in that other place, where the man must be who invented the instrument of torture." [1]

Those of you who have had the pleasure of seeing Dr. Fillebrown extract with the key know that in his hands, in selected cases, it is an efficient instrument.

One of the early writers says, "When teeth bleed too freely after extraction a little lint is pressed into the socket and a cushion of rags is crowded into the space on which the patient is instructed to bite." [2]

I have here some impression trays which look as if they had seen service, and no doubt are hand-made.

Dr. Samuel A. Bemis, 32 School Street, was practising as early as 1819. I have not been able to find much about him. He was one of the first to fill root-canals in this country.

We now come to a man who has certainly earned the high place in his profession which he holds. Nathaniel Cooley Keep was born in Longmeadow, Mass., December 23, 1800. He probably inherited his mechanical skill from his father, Samuel Keep, who had great ingenuity, and very often would do with his own hands operations of the wheelwright, blacksmith, and carpenter. From childhood his career seemed to be marked out for a mechanical pursuit on account of his skill in the use of tools, and at the age of fifteen, with the limited education of a village school, he went to Newark,

---

[1] O. W. Holmes, The Claims of Dentistry.

[2] Benjamin James.

N. J., where he was apprenticed to John Taylor, a manufacturing jeweller. But after five years' apprenticeship he returned to Longmeadow with the determination to go to Boston and give his time and energy to dentistry. His years of apprenticeship in Newark had afforded him good training. He was often obliged to make his own tools and to discover for himself the best way of performing many delicate and difficult tasks. His practical experience in working with metals which he had obtained in the jewelry business was a great help to him. Dr. Keep early recognized the truth that a general acquaintance with medical science was necessary in order to reach a high eminence in dentistry, and under this conviction he attended the regular course of lectures at Harvard Medical College, and at the same time kept up his practice of dentistry. He took his medical degree in 1827. The practical training of his professional work was received from Dr. John Randall, of Boston, who, as was common in those days, united some practice of dentistry with the general practice of a physician.

During Dr. Keep's many years of practice there was nothing narrow or exclusive in his course respecting the progress of his art, as there was nothing ungenerous in his character. His spirit in this respect is well expressed in the following extract from an address delivered by him before the Massachusetts Dental Society, of which he was the first president, on "The Aims and Duties of the Dental Profession," and published in 1865. He said, "We owe it to ourselves to make ours a liberal profession. Without enumerating all that such a profession comprises, we may safely say that it requires those of its members, who have through their own efforts, or the teachings of those who have preceded them, made improvements in dental science to perpetuate these improvements for the benefit of succeeding generations, and under no circumstances whatever to desire or even consent that their discoveries shall live and die with themselves."

It was largely through Dr. Keep's efforts that the Dental School of Harvard University was established in 1868. Its aim was declared to be "to raise the standard of dental education by giving thorough instruction in all branches of science and art required by the dental practitioner." The connection of this school with the university furnished the profession a guarantee that its standard would be high, as it must necessarily be to keep pace with the other departments. Dr. Keep was dean of the first faculty.

They treated over one thousand patients there during the first year. In 1870 Dr. Keep received the honorary degree of Doctor of Dental Medicine from Harvard College. As most of you are familiar with the noted Parkman and Webster murder trial, in which Dr. Keep was the principal witness, I will only mention it here. His life was given to relieve human suffering. He contributed much aid in building up a liberal profession, and, at peace with all, his useful life was closed on the 11th of March, 1875. I have here a beautiful specimen of Dr. Keep's work, a set of carved teeth.

I will now show you a set of ivory points designed by the late Dr. G. T. Moffatt. I have been told that they were made to insert gold fillings. Here is a steel instrument which he designed for finishing gold fillings.

Daniel Harwood, M.D., who was born in Barre, Mass., March 21, 1801, determined very young in life to make the profession and practice of medicine his study and effort. He commenced to practise in Northampton, Mass., and afterwards he entered the Medical School at Brunswick College, where he graduated. He made dentistry his specialty, and settled for a short time in Portland, Me., where he was associated with Dr. Prentiss. In 1829 he came to Boston, where he located permanently, and was associated with Dr. Lane, then with Dr. Josiah Tucker.

In a short time Dr. Harwood's practice was larger than he could personally attend to. In fact, he is said to have reached the highest professional standing at that time. After 1873 he gradually retired from the active duties of his profession, and for the last six years before his death, which occurred in October, 1881, he gave no time to it. The marked characteristics of Dr. Harwood were energy of purpose, perseverance, and strict integrity.

Let me now call your attention to Dr. Joshua Tucker, who was born in Winchendon, Mass., August 7, 1800. Here he spent his boyhood days, and at the age of eighteen he went in business. But in the course of a few years he turned his attention to dentistry. He commenced his study with Dr. D. C. Ambler, of Columbia, S. C., and later went with C. Starr Brewster, of Charleston, meanwhile attending lectures at the South Carolina Medical College. He completed his studies here, then went to Havana, Cuba, where he practised his profession until driven away several years later by yellow fever. In 1833 he came to Boston, and was associated with Dr. Daniel Harwood, and the name of Harwood & Tucker soon became well-known both in this country and in Europe. Always cheerful, happy, and good-natured, he naturally had a long chain of faithful and admiring friends, to whom he was strongly attached, until death came, at the age of eighty-one years and three months. He had been a member of the Massachusetts Medical Society since 1838, and was president and honorary member of several professional societies, one of which was the Odontological Society of Great Britain.

I will pass around some more instruments which may prove interesting to some.

This porcelain artificial nose, made by Willard Codman fifty-five or sixty years ago, is a duplicate of a practical case. I think if you study it a minute you will see that some of these early dentists did artistic work.

Dr. Alonzo F. Preston, who has practised dentistry in Boston for sixty-five years, and is now about ninety-two years of age, has kindly given me much data for this article. He says that when he began dental work he was a jeweller, and at that time there were only four or five other men practising the profession in Boston,—Drs. Bemis, Harwood, N. C. Keep, and Tucker.

When a patient came into Dr. Preston's office, he would examine the teeth and see what there was to be done, then give another appointment, and in the mean time he would make such instruments as were necessary to do the operation.

I will pass around the first two instruments made and used by him, also some of the last he made. I have here a gold bridge which he made in 1839, which the patient wore nine years.

Dr. Preston said that previous to 1827 he could not get his own front teeth filled in Boston. The operation at that time was not considered practical.

In closing, I wish to say that if I have made any errors in compiling this article I shall be glad to have them corrected. I have tried to give you a few facts, although they are disconnected. If anything I have said has interested you so that you will "observe, compare, reflect, record," it will give me great pleasure.

Gentlemen, I thank you for your attention.

